# Supplementary material for: Therapeutic senescence via GPCR activation in synovial fibroblasts facilitates resolution of arthritis
Source: Nat Commun. 2020 Feb 6;11:745. doi: 10.1038/s41467-020-14421-x (PMC7005314; doi:10.1038/s41467-020-14421-x)
Supplement: Supplementary file 1 — Supplementary Information [file 41467_2020_14421_MOESM1_ESM.pdf]

Supplementary Information

Therapeutic senescence via GPCR activation in synovial fibroblasts facilitates resolution of arthritis

Montero-Melendez T *et al.*

Supplementary Table 1. Summary of clinical details of patients (n=20) at the time of fibroblasts sample collection (mean±SD).

	**Patients**
**Age** -years-	50 ± 14.55
**Female, n (%)**	10 (50%)
**ESR** -mm/h-	29 ± 22.8
**CRP** -mg/l-	24 ± 23.4
**DAS28ESR**	5 ± 1.5
**Symptom duration** -weeks-	342 ± 559
**IgM-RF$^+$, n (%)**	9 (45%)
**CCP$^+$, n (%)**	5 (25%)
**Ethnicity, n (%)**	White British: 17 (85%)<br>Caribbean: 2 (10%)<br>Indian: 1 (5%)

Supplementary Table 2. Primers used in this study.

PCR primers		
Hs_ADAMTS2_1_SG QuantiTect Primer Assay	QIAGEN	QT00020083
Hs_AGRP_1_SG QuantiTect Primer Assay	QIAGEN	QT00204477
Hs_ASIP_1_SG QuantiTect Primer Assay	QIAGEN	QT00061509
Hs_BMP2_1_SG QuantiTect Primer Assay	QIAGEN	QT00012544
Hs_CD74_1_SG QuantiTect Primer Assay	QIAGEN	QT00059402
Hs_CDH11_1_SG QuantiTect Primer Assay	QIAGEN	QT00019418
Hs_CDKN1A_1_SG QuantiTect Primer Assay	QIAGEN	QT00062090
Hs_CDKN2A_1_SG QuantiTect Primer Assay	QIAGEN	QT00089964
Hs_COL3A1_1_SG QuantiTect Primer Assay	QIAGEN	QT00058233
Hs_COL3A1_1_SG QuantiTect Primer Assay	QIAGEN	QT00058233
Hs_COL5A1_1_SG QuantiTect Primer Assay	QIAGEN	QT00044527
Hs_CTSK_1_SG QuantiTect Primer Assay	QIAGEN	QT00093856
Hs_CTSL_1_SG QuantiTect Primer Assay	QIAGEN	QT01664978
Hs_DMKN_1_SG QuantiTect Primer Assay	QIAGEN	QT00057897
Hs_FGF2_1_SG QuantiTect Primer Assay	QIAGEN	QT00047579
Hs_FTL_1_SG QuantiTect Primer Assay	QIAGEN	QT00055860
Hs_GLB1_1_SG QuantiTect Primer Assay	QIAGEN	QT00066206
Hs_GPBAR1_1_SG QuantiTect Primer Assay	QIAGEN	QT00209594
Hs_GHSR_1_SG QuantiTect Primer Assay	QIAGEN	QT00040901
Hs_HMGCR_1_SG QuantiTect Primer Assay	QIAGEN	QT00004081
Hs_HMOX1_1_SG QuantiTect Primer Assay	QIAGEN	QT00092645
Hs_HPRT1_1_SG QuantiTect Primer Assay	QIAGEN	QT00059066
Hs_HRAS_2_SG QuantiTect Primer Assay	QIAGEN	QT01668338
Hs_HSD3B7_2_SG QuantiTect Primer Assay	QIAGEN	QT02316300
Hs_ICAM1_1_SG QuantiTect Primer Assay	QIAGEN	QT00074900
Hs_INSIG1_1_SG QuantiTect Primer Assay	QIAGEN	QT00090314
Hs_LDLR_1_SG QuantiTect Primer Assay	QIAGEN	QT00045864
Hs_MC1R_1_SG QuantiTect Primer Assay	QIAGEN	QT01004241
Hs_MC2R_2_SG QuantiTect Primer Assay	QIAGEN	QT01155007
Hs_MC3R_1_SG QuantiTect Primer Assay	QIAGEN	QT00209895
Hs_MC4R_1_SG QuantiTect Primer Assay	QIAGEN	QT00245595
Hs_MC5R_1_SG QuantiTect Primer Assay	QIAGEN	QT00211960
Hs_MMP11_1_SG QuantiTect Primer Assay	QIAGEN	QT00024031

Hs_MRAP_1_SG QuantiTect Primer Assay	QIAGEN	QT00103866
Hs_MRAP2_1_SG QuantiTect Primer Assay	QIAGEN	QT00493150
Hs_PCSK1_1_SG QuantiTect Primer Assay	QIAGEN	QT00013853
Hs_PCSK2_1_SG QuantiTect Primer Assay	QIAGEN	QT00054754
Hs_POMC_1_SG QuantiTect Primer Assay	QIAGEN	QT00001204
Hs_POSTN_1_SG QuantiTect Primer Assay	QIAGEN	QT00023800
Hs_PTEN_1_SG QuantiTect Primer Assay	QIAGEN	QT00086933
Hs_TNC_1_SG QuantiTect Primer Assay	QIAGEN	QT00024409
Hs_TP53_1_SG QuantiTect Primer Assay	QIAGEN	QT00060235
MC1R-Forward: 5'-GAAGAACTGTGGGGACCTGG-3'	Sigma-Aldrich	In-house designed
MC1R-Reverse: 5'-GGGTCACACAGGAACCAGAC-3'	Sigma-Aldrich	In-house designed

Supplementary Table 3. Extended information on genes identified by RNAseq.

Symbol	Gene name	FC	Refseq ID	Locus
**qPCR validation (Related to Fig3A,B)**				
**ADAMTS2**	ADAM metallopeptidase with thrombospondin type 1 motif 2	0.79	NM_014244	chr5 : 179110850 - 179345430
**CD74**	CD74 molecule, major histocompatibility complex, class II invariant chain	0.74	NM_004355	chr5 : 150401636 - 150412936
**CDH11**	cadherin 11, type 2, OB-cadherin (osteoblast)	0.84	NM_001797	chr16 : 64943752 - 65122137
**CDKN2A**	cyclin-dependent kinase inhibitor 2A	1.09	NM_000077	chr9 : 21967751 - 21975133
**COL3A1**	collagen, type III, alpha 1	0.65	NM_000090	chr2 : 188974372 - 189012746
**COL5A1**	collagen, type V, alpha 1	0.72	NM_000093	chr9 : 134641804 - 134844842
**CTSK**	cathepsin K	1.99	NM_000396	chr1 : 150796207 - 150808441
**DMKN**	dermokine	1.79	NM_001035516	chr19 : 35497216 - 35501911
**HMGCR**	3-hydroxy-3-methylglutaryl-CoA reductase	1.53	NM_001130996	chr5 : 75337167 - 75362101
**HMOX1**	heme oxygenase 1	1.58	NM_002133	chr22 : 35381066 - 35394214
**HRAS**	Harvey rat sarcoma viral oncogene homolog	1.12	NM_001130442	chr11 : 532241 - 535567
**HSD3B7**	hydroxy-delta-5-steroid dehydrogenase, 3 beta- and steroid delta-isomerase 7	1.41	NM_001142777	chr16 : 30985197 - 30989152
**INSIG1**	insulin induced gene 1	1.98	NM_005542	chr7 : 155297775 - 155310235
**LDLR**	low density lipoprotein receptor	2.04	NM_001195799	chr19 : 11089361 - 11133829
**MMP11**	matrix metallopeptidase 11	1.29	NM_005940	chr22 : 23772818 - 23784316
**POSTN**	periostin, osteoblast specific factor	0.80	NM_006475	chr13 : 37562581 - 37598844
**PTEN**	phosphatase and tensin homolog	0.89	NM_000314	chr10 : 87863437 - 87971930
**TNC**	tenascin C	0.75	NM_002160	chr9 : 115019575 - 115118257

Senescence profile (Related to Figure 3C)				
**BAG1**	BCL2 associated athanogene 1	1.12	NM_001172415	chr9 : 33252470 - 33264761
**BCL2**	B-cell CLL/lymphoma 2	1.58	NM_000633	chr18 : 63123345 - 63319380
**BCL2L1**	BCL2-like 1	1.39	NM_001191	chr20 : 31664451 - 31723098
**CCND2**	cyclin D2	0.91	NM_001759	chr12 : 4273735 - 4305356
**CCNI**	cyclin I	0.91	NM_006835	chr4 : 77048020 - 77075972
**CDC7**	cell division cycle 7	0.79	NM_003503	chr1 : 91500846 - 91525764
**CDK6**	cyclin-dependent kinase 6	0.83	NM_001259	chr7 : 92604920 - 92833917
**CDK5R1**	cyclin-dependent kinase 5, regulatory subunit 1 (p35)	0.81	NM_003885	chr17 : 32487086 - 32491253
**CDKN1A**	cyclin-dependent kinase inhibitor 1A (p21, Cip1)	1.39	NM_001291549	chr6 : 36676536 - 36687339
**CDKN2A**	cyclin-dependent kinase inhibitor 2A (p16)	1.09	NM_000077	chr9 : 21967751 - 21975133
**CDKN2B**	cyclin-dependent kinase inhibitor 2B (p15, inhibits CDK4)	1.29	NM_004936	chr9 : 22002902 - 22009313
**E2F7**	E2F transcription factor 7	1.43	NM_203394	chr12 : 77021245 - 77065580
**PTEN**	phosphatase and tensin homolog	0.89	NM_000314	chr10 : 87863437 - 87971930
**RBBP9**	retinoblastoma binding protein 9	1.13	NM_006606	chr20 : 18486540 - 18497243

Extracellular matrix (Related to Figure 3F)				
**ADAMTS1**	ADAM metallopeptidase with thrombospondin type 1 motif 1	0.71	NM_006988	chr21 : 26836286 - 26845409
**ADAMTS12**	ADAM metallopeptidase with thrombospondin type 1 motif 12	0.80	NM_030955	chr5 : 33527180 - 33892019
**ADAMTS15**	ADAM metallopeptidase with thrombospondin type 1 motif 15	0.67	NM_139055	chr11 : 130448973 - 130476644
**ADAMTS16**	ADAM metallopeptidase with thrombospondin type 1 motif 16	0.77	NM_139056	chr5 : 5140329 - 5320299
**ADAMTS2**	ADAM metallopeptidase with thrombospondin type 1 motif 2	0.79	NM_014244	chr5 : 179110850 - 179345430
**ADAMTS3**	ADAM metallopeptidase with thrombospondin type 1 motif 3	0.64	NM_014243	chr4 : 72280968 - 72568799
**B4GAT1**	beta-1,4-glucuronyltransferase 1	0.90	NM_006876	chr11 : 66345371 - 66347690
**COL1A1**	collagen, type I, alpha 1	0.80	NM_000088	chr17 : 50184095 - 50201639
**COL3A1**	collagen, type III, alpha 1	0.65	NM_000090	chr2 : 188974372 - 189012746

**COL5A1**	collagen, type V, alpha 1	0.72	NM_000093	chr9 : 134641804 - 134844842
**COL5A3**	collagen, type V, alpha 3	0.67	NM_015719	chr19 : 9959560 - 10010471
**COL6A1**	collagen, type VI, alpha 1	0.78	NM_001848	chr21 : 45981748 - 46005049
**COL14A1**	collagen, type XIV, alpha 1	0.74	NM_021110	chr8 : 120125107 - 120372034
**DPP4**	dipeptidyl-peptidase 4	1.27	NM_001935	chr2 : 161992244 - 162074542
**GALNT12**	polypeptide N-acetylgalactosaminyltransferase 12	1.53	NM_024642	chr9 : 98807698 - 98850081
**GLT8D1**	glycosyltransferase 8 domain containing 1	0.90	NM_152932	chr3 : 52694483 - 52703698
**LAMA2**	laminin subunit alpha 2	0.87	NM_001079823	chr6 : 128883140 - 129516565
**LARGE**	like-glycosyltransferase	1.21	NM_004737	chr22 : 33273075 - 33920428
**LOXL2**	lysyl oxidase like 2	0.86	NM_002318	chr8 : 23296896 - 23404209
**MME**	membrane metallo-endopeptidase	1.76	NM_007288	chr3 : 155080163 - 155183729
**MMP11**	matrix metallopeptidase 11	1.29	NM_005940	chr22 : 23772818 - 23784316
**MMP3**	matrix metallopeptidase 3	1.48	NM_002422	chr11 : 102835796 - 102843689

Cell cycle regulation (Related to Figure 3F)

**CCND2**	cyclin D2	0.91	NM_001759	chr12 : 4273735 - 4305356
**CDC7**	cell division cycle 7	0.79	NM_003503	chr1 : 91500846 - 91525764
**CDIP1**	cell death-inducing p53 target 1	1.17	NM_013399	chr16 : 4510675 - 4538815
**CDK6**	cyclin-dependent kinase 6	0.83	NM_001259	chr7 : 92604920 - 92833917
**CNTRL**	centriolin	0.84	NM_007018	chr9 : 121088295 - 121177608
**FIGN**	fidgetin	0.88	NM_018086	chr2 : 163607607 - 163736003
**G2E3**	G2/M-phase specific E3 ubiquitin protein ligase	0.93	NM_017769	chr14 : 30559122 - 30620061
**GAS1**	growth arrest specific 1	0.69	NM_002048	chr9 : 86944361 - 86947189
**ING1**	inhibitor of growth family member 1	0.85	NM_005537	chr13 : 110715011 - 110721074
**RBBP9**	retinoblastoma binding protein 9	1.13	NM_006606	chr20 : 18486540 - 18497243

Lysosome markers (Related to Figure 3F)				
**ARSG**	arylsulfatase G	1.36	NM_001267727	chr17 : 68291494 - 68420859
**ATP6V0A1**	ATPase, H+ transporting, lysosomal V0 subunit a1	1.22	NM_005177	chr17 : 42458843 - 42522579
**ATP6V1H**	ATPase, H+ transporting, lysosomal 50/57kDa, V1 subunit H	1.25	NM_213620	chr8 : 53715542 - 53843042
**CTSK**	cathepsin K	1.99	NM_000396	chr1 : 150796207 - 150808441
**CTSL**	cathepsin L	1.23	NM_001912	chr9 : 87726058 - 87731469
**CTSS**	cathepsin S	1.33	NM_001199739	chr1 : 150730195 - 150765957
**LIPA**	lipase A, lysosomal acid, cholesterol esterase	1.19	NM_000235	chr10 : 89213568 - 89252039
**M6PR**	mannose-6-phosphate receptor (cation dependent)	1.21	NM_001207024	chr12 : 8940360 - 8949761
**NPC1**	Niemann-Pick disease, type C1	1.56	NM_000271	chr18 : 23531498 - 23586617
**NPC2**	Niemann-Pick disease, type C2	1.25	NM_006432	chr14 : 74479939 - 74493381

Synovial Fibroblast aggressiveness (Related to Figure 3G)				
**CCL2**	chemokine (C-C motif) ligand 2	0.77	NM_002982	chr17 : 34255276 - 34257201
**CD74**	CD74 molecule, major histocompatibility complex, class II invariant chain	0.74	NM_004355	chr5 : 150401636 - 150412936
**CXCL16**	chemokine (C-X-C motif) ligand 16	0.85	NM_022059	chr17 : 4733528 - 4739928
**IL17RC**	interleukin 17 receptor C	0.92	NM_001203263	chr3 : 9917073 - 9933621
**IL18R1**	interleukin 18 receptor 1	0.75	NM_003855	chr2 : 102356282 - 102398775
**ITGA9**	integrin subunit alpha 9	0.65	NM_002207	chr3 : 37452321 - 37819790
**ITGA11**	integrin subunit alpha 11	0.87	NM_001004439	chr15 : 68301703 - 68432153
**MSTN**	myostatin	0.50	NM_005259	chr2 : 190055699 - 190062729
**PTGS2**	prostaglandin-endoperoxide synthase 2	0.82	NM_000963	chr1 : 186671811 - 186680427
**SENP1**	SUMO1/sentrin specific peptidase 1	0.90	NM_001267595	chr12 : 48042897 - 48105992
**THBS4**	thrombospondin 4	0.76	NM_001306212	chr5 : 79991295 - 80083284
**TNC**	tenascin C	0.75	NM_002160	chr9 : 115019575 - 115118257
**VCAM1**	vascular cell adhesion molecule 1	0.65	NM_080682	chr1 : 100719639 - 100739046

**VEGFA**	vascular endothelial growth factor A	0.85	NM_001204385	chr6 : 43770208 - 43786486

Identified from Cell Age (Related to Figure 3H)

**ACLY**	ATP citrate lyase	1.15	NM_001303275	chr17 : 41866916 - 41919022
**BHLHE40**	basic helix-loop-helix family member e40	1.17	NM_003670	chr3 : 4979411 - 4985180
**BTG3**	BTG family member 3	1.16	NM_006806	chr21 : 17593649 - 17612950
**CCND1**	cyclin D1	1.27	NM_053056	chr11 : 69641104 - 69654474
**CDK6**	cyclin-dependent kinase 6	0.83	NM_001259	chr7 : 92604920 - 92833917
**CDKN1A**	cyclin-dependent kinase inhibitor 1A (p21, Cip1)	1.39	NM_001291549	chr6 : 36676536 - 36687339
**CDKN1B**	cyclin-dependent kinase inhibitor 1B (p27, Kip1)	0.86	NM_004064	chr12 : 12717269 - 12722382
**CDKN2A**	cyclin-dependent kinase inhibitor 2A (p16)	1.09	NM_000077	chr9 : 21967751 - 21975133
**CDKN2B**	cyclin-dependent kinase inhibitor 2B (p15, inhibits CDK4)	1.29	NM_004936	chr9 : 22002902 - 22009313
**CPEB1**	cytoplasmic polyadenylation element binding protein 1	1.28	NM_001288819	chr15 : 82543200 - 82647605
**CXCL1**	chemokine (C-X-C motif) ligand 1	0.53	NM_001511	chr4 : 73869391 - 73871302
**DHCR24**	24-dehydrocholesterol reductase	1.48	NM_014762	chr1 : 54849626 - 54887248
**DUSP3**	dual specificity phosphatase 3	1.19	NM_004090	chr17 : 43766120 - 43779000
**HDAC4**	histone deacetylase 4	0.89	NM_006037	chr2 : 239048167 - 239400949
**HRAS**	Harvey rat sarcoma viral oncogene homolog	1.12	NM_001130442	chr11 : 532241 - 535567
**HSPB2**	heat shock protein family B (small) member 2	1.23	NM_001541	chr11 : 111912735 - 111914093
**ID1**	inhibitor of DNA binding 1, dominant negative helix-loop-helix protein	0.79	NM_002165	chr20 : 31605282 - 31606514
**ING1**	inhibitor of growth family member 1	0.85	NM_005537	chr13 : 110715011 - 110721074
**KDM5B**	lysine (K)-specific demethylase 5B	0.91	NM_006618	chr1 : 202725184 - 202809470
**LIMK1**	LIM domain kinase 1	1.26	NM_001204426	chr7 : 74093155 - 74122525
**MAP2K3**	mitogen-activated protein kinase kinase 3	1.15	NM_002756	chr17 : 21288035 - 21315239
**MVK**	mevalonate kinase	1.53	NM_000431	chr12 : 109573694 - 109597270
**NOTCH3**	notch 3	0.84	NM_000435	chr19 : 15159632 - 15200981

**NTN4**	netrin 4	1.13	NM_021229	chr12 : 95657806 - 95790758
**P3H1**	prolyl 3-hydroxylase 1	0.84	NM_022356	chr1 : 42746334 - 42767084
**PRKCH**	protein kinase C, eta	0.81	NM_006255	chr14 : 61321442 - 61550980
**PSMD14**	proteasome 26S subunit, non-ATPase 14	1.13	NM_005805	chr2 : 161308274 - 161411717
**RNASEL**	ribonuclease L (2',5'-oligoisoadenylate synthetase-dependent)	0.88	NM_021133	chr1 : 182573633 - 182589259
**SENP1**	SUMO1/sentrin specific peptidase 1	0.90	NM_001267595	chr12 : 48042897 - 48105992
**SENP7**	SUMO1/sentrin specific peptidase 7	0.91	NM_001077203	chr3 : 101324188 - 101513241
**SIX1**	SIX homeobox 1	0.89	NM_005982	chr14 : 60644698 - 60649437
**SMG1**	SMG1 phosphatidylinositol 3-kinase-related kinase	0.93	NM_015092	chr16 : 18804852 - 18926404
**SREBF1**	sterol regulatory element binding transcription factor 1	1.77	NM_001005291	chr17 : 17811348 - 17837011
**TXN**	thioredoxin	1.10	NM_003329	chr9 : 110243811 - 110256640
**VEGFA**	vascular endothelial growth factor A	0.85	NM_001204385	chr6 : 43770208 - 43786486
**WNT2**	wingless-type MMTV integration site family member 2	0.56	NR_024047	chr7 : 117276630 - 117323289
**ZFP36**	ZFP36 ring finger protein	0.92	NM_003407	chr19 : 39406846 - 39409412

Identified from TiRe (Related to Figure 3H)

**ADAMTS1**	ADAM metallopeptidase with thrombospondin type 1 motif 1	0.71	NM_006988	chr21 : 26836286 - 26845409
**ADRA2A**	adrenoceptor alpha 2A	0.75	NM_000681	chr10 : 111077031 - 111080904
**AHR**	aryl hydrocarbon receptor	0.75	NM_001621	chr7 : 17298651 - 17346151
**ANGPTL4**	angiopoietin like 4	0.76	NR_104213	chr19 : 8364126 - 8374375
**ARHGAP1**	Rho GTPase activating protein 1	1.14	NM_004308	chr11 : 46677074 - 46700665
**B4GALNT1**	beta-1,4-N-acetyl-galactosaminyl transferase 1	1.20	NM_001276469	chr12 : 57629330 - 57633239
**CCL2**	chemokine (C-C motif) ligand 2	0.77	NM_002982	chr17 : 34255276 - 34257201
**CD151**	CD151 molecule (Raph blood group)	1.07	NM_139030	chr11 : 832951 - 838835
**CDKN1B**	cyclin-dependent kinase inhibitor 1B (p27, Kip1)	0.86	NM_004064	chr12 : 12717269 - 12722382
**COL1A1**	collagen, type I, alpha 1	0.80	NM_000088	chr17 : 50184095 - 50201639

**CXXC5**	CXXC finger protein 5	0.88	NM_001317204	chr5 : 139648924 - 139683885
**ESR1**	estrogen receptor 1	1.16	NM_000125	chr6 : 151807678 - 152103273
**FGF2**	fibroblast growth factor 2 (basic)	1.21	NM_002006	chr4 : 122826707 - 122898235
**HMOX1**	heme oxygenase 1	1.58	NM_002133	chr22 : 35381066 - 35394214
**ICAM1**	intercellular adhesion molecule 1	1.27	NM_000201	chr19 : 10270840 - 10286615
**ITGA11**	integrin subunit alpha 11	0.87	NM_001004439	chr15 : 68301703 - 68432153
**ITGA9**	integrin subunit alpha 9	0.65	NM_002207	chr3 : 37452321 - 37819790
**LSP1**	lymphocyte-specific protein 1	0.86	NM_001013255	chr11 : 1870923 - 1892263
**MRC2**	mannose receptor, C type 2	0.87	NM_006039	chr17 : 62627400 - 62693601
**MSTN**	myostatin	0.50	NM_005259	chr2 : 190055699 - 190062729
**NFIC**	nuclear factor I/C (CCAAT-binding transcription factor)	0.86	NM_005597	chr19 : 3366566 - 3469217
**NID1**	nidogen 1	0.82	NM_002508	chr1 : 235975831 - 236065181
**NR4A1**	nuclear receptor subfamily 4 group A member 1	1.16	NM_001202233	chr12 : 52022831 - 52059507
**PGF**	placental growth factor	0.66	NM_001207012	chr14 : 74941829 - 74955764
**PLAUR**	plasminogen activator, urokinase receptor	1.16	NM_001005376	chr19 : 43646094 - 43670346
**POSTN**	periostin, osteoblast specific factor	0.80	NM_006475	chr13 : 37562581 - 37598844
**PPARA**	peroxisome proliferator-activated receptor alpha	0.92	NM_001001928	chr22 : 46150595 - 46243756
**PPARG**	peroxisome proliferator-activated receptor gamma	1.25	NM_138711	chr3 : 12288936 - 12434356
**PRKCH**	protein kinase C, eta	0.81	NM_006255	chr14 : 61321442 - 61550980
**PTEN**	phosphatase and tensin homolog	0.89	NM_000314	chr10 : 87863437 - 87971930
**PTGS2**	prostaglandin-endoperoxide synthase 2	0.82	NM_000963	chr1 : 186671811 - 186680427
**SNAI2**	snail family zinc finger 2	0.80	NM_003068	chr8 : 48917680 - 48921440
**TGFB3**	transforming growth factor beta 3	0.83	NM_003239	chr14 : 75958096 - 75982022
**THBD**	thrombomodulin	1.55	NM_000361	chr20 : 23045632 - 23049664
**TNFRSF1A**	tumor necrosis factor receptor superfamily member 1A	0.89	NM_001065	chr12 : 6328756 - 6342117
**VAV3**	vav guanine nucleotide exchange factor 3	1.36	NM_006113	chr1 : 107571159 - 107964923

**VEGFA**	vascular endothelial growth factor A	0.85	NM_001204385	chr6 : 43770208 - 43786486
**VTN**	vitronectin	0.70	NM_000638	chr17 : 28367277 - 28370352

Notch pathway (Related to Figure 4)

**CREBBP**	CREB binding protein	0.92	NM_004380	chr16 : 3725054 - 3880120
**DTX3L**	deltex 3 like, E3 ubiquitin ligase	0.89	NM_138287	chr3 : 122564337 - 122575202
**DTX4**	deltex 4, E3 ubiquitin ligase	0.64	NM_015177	chr11 : 59172338 - 59208587
**EP300**	E1A binding protein p300	0.90	NM_001429	chr22 : 41092609 - 41180077
**HES1**	hes family bHLH transcription factor 1	0.81	NM_005524	chr3 : 194136141 - 194138612
**JAG1**	jagged 1	0.78	NM_000214	chr20 : 10637683 - 10674046
**MAML2**	mastermind like transcriptional coactivator 2	0.87	NM_032427	chr11 : 95976592 - 96343180
**MAML3**	mastermind like transcriptional coactivator 3	0.79	NM_018717	chr4 : 139716390 - 140154079
**MIB1**	mindbomb E3 ubiquitin protein ligase 1	0.93	NM_020774	chr18 : 21741328 - 21870957
**NCOR2**	nuclear receptor corepressor 2	0.93	NM_001206654	chr12 : 124324410 - 124567464
**NOTCH3**	notch 3	0.84	NM_000435	chr19 : 15159632 - 15200981

Cholesterol pathway (Related to Figure 5)

**ABCA1**	ATP binding cassette subfamily A member 1	1.26	NM_005502	chr9 : 104781002 - 104928246
**DHCR24**	24-dehydrocholesterol reductase	1.48	NM_014762	chr1 : 54849626 - 54887248
**DHCR7**	7-dehydrocholesterol reductase	1.97	NM_001360	chr11 : 71434410 - 71448431
**FDFT1**	farnesyl-diphosphate farnesyltransferase 1	1.25	NM_001287744	chr8 : 11795572 - 11839309
**FDPS**	farnesyl diphosphate synthase	1.57	NM_002004	chr1 : 155308747 - 155320666
**HMGCR**	3-hydroxy-3-methylglutaryl-CoA reductase	1.53	NM_001130996	chr5 : 75337167 - 75362101
**HMGCS1**	3-hydroxy-3-methylglutaryl-CoA synthase 1	1.95	NM_001098272	chr5 : 43287469 - 43313512
**HSD3B7**	hydroxy-delta-5-steroid dehydrogenase, 3 beta- and steroid delta-isomerase 7	1.41	NM_001142777	chr16 : 30985197 - 30989152
**IDI1**	isopentenyl-diphosphate delta isomerase 1	1.57	NM_004508	chr10 : 1039419 - 1049366

**INSIG1**	insulin induced gene 1	1.98	NM_005542	chr7 : 155297775 - 155310235
**LDLR**	low density lipoprotein receptor	2.04	NM_001195799	chr19 : 11089361 - 11133829
**LSS**	lanosterol synthase (2,3-oxidosqualene-lanosterol cyclase)	1.27	NM_001145437	chr21 : 46189123 - 46228824
**MVK**	mevalonate kinase	1.53	NM_000431	chr12 : 109573694 - 109597270
**NPC1**	Niemann-Pick disease, type C1	1.56	NM_000271	chr18 : 23531498 - 23586617
**NPC2**	Niemann-Pick disease, type C2	1.25	NM_006432	chr14 : 74479939 - 74493381
**NSDHL**	NAD(P) dependent steroid dehydrogenase-like	1.21	NM_001129765	chrX : 152830966 - 152869363
**PCSK9**	proprotein convertase subtilisin/kexin type 9	2.85	NM_174936	chr1 : 55039475 - 55064853
**SQLE**	squalene epoxidase	1.48	NM_003129	chr8 : 124998477 - 125022283
**SREBF1**	sterol regulatory element binding transcription factor 1	1.77	NM_001005291	chr17 : 17811348 - 17837011
**SREBF2**	sterol regulatory element binding transcription factor 2	1.37	NR_103834	chr22 : 41833078 - 41907308

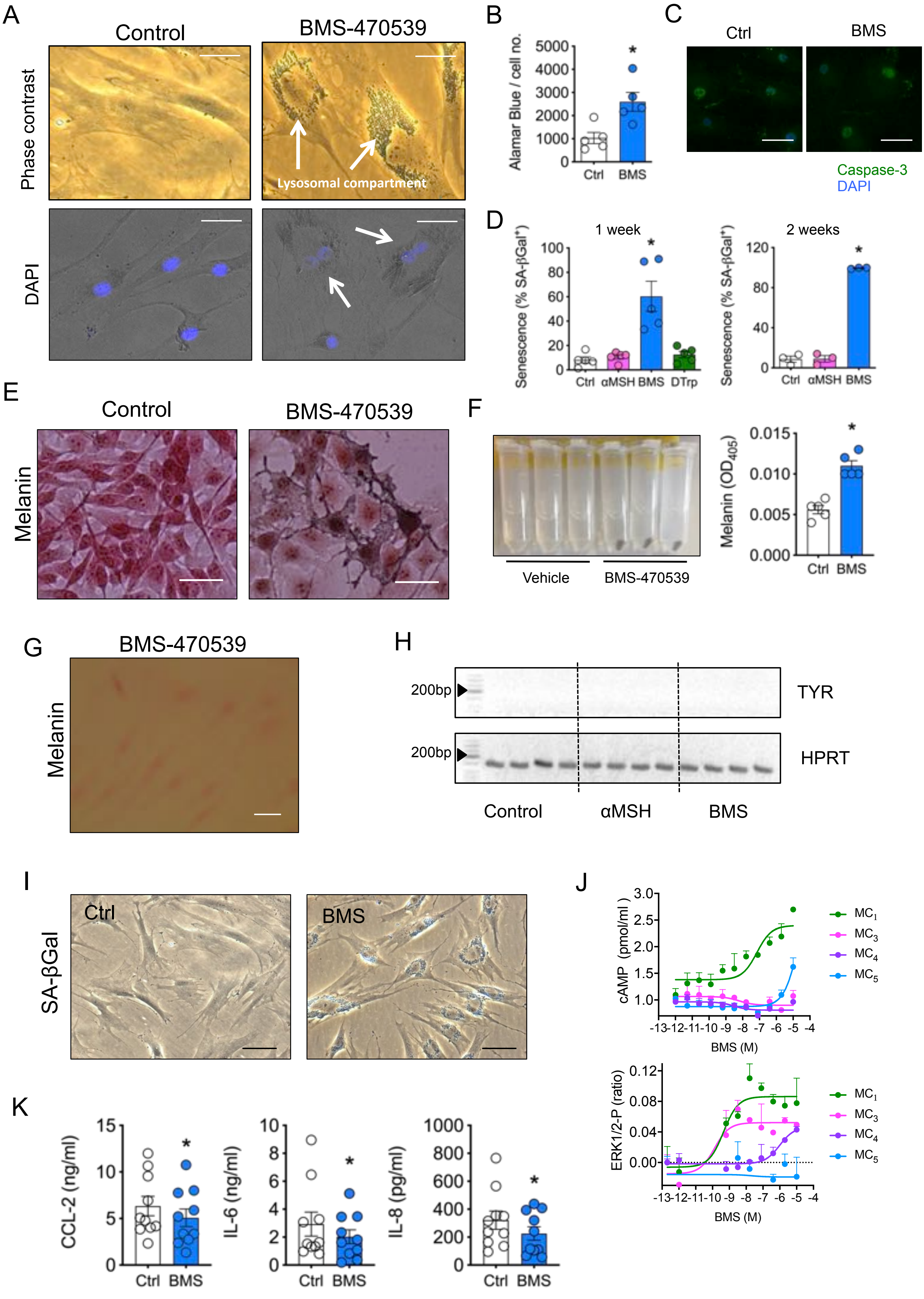
A
Control
BMS-470539
Phase contrast
Lysosomal compartment
DAPI
B
Alamar Blue / cell no.
5000
4000
3000
2000
1000
0
Ctrl
BMS
*
C
Ctrl
BMS
Caspase-3
DAPI
D
1 week
2 weeks
Senescence (% SA-βGal+)
100
80
60
40
20
0
120
Ctrl
αMSH
BMS
DTrp
*
E
Control
BMS-470539
Melanin
F
Vehicle
BMS-470539
Melanin (OD405)
0.015
0.010
0.005
0.000
Ctrl
BMS
G
BMS-470539
Melanin
H
200bp
TYR
HPRT
Control
αMSH
BMS
I
SA-βGal
Ctrl
BMS
J
cAMP (pmol/ml)
3.0
2.5
2.0
1.5
1.0
-13-12-11-10 -9 -8 -7 -6 -5 -4
BMS (M)
MC1
MC3
MC4
MC5
ERK1/2-P (ratio)
0.12
0.08
0.04
0.00
K
CCL-2 (ng/ml)
15
10
5
0
IL-6 (ng/ml)
10
8
6
4
2
0
IL-8 (pg/ml)
1000
800
600
400
200
0
Ctrl
BMS
*

Supplementary Figure 1. Effect of BMS on fibroblast senescence, melanin production and signalling.

(A) Images of SF (control or treated with 1µM BMS) showing expansion of lysosomal compartment and presence of bi-nucleated cells (DAPI staining). Scale bars indicate 100µm.

(B) SF were treated with BMS (1µM) for 7 days. Alamar blue assay was used as an indicator of cell health and metabolic activity. Data represent mean values ±SE (n=5, Student's t-test vs. control).

(C) Cleaved caspase-3 expression was assessed by immunofluorescence on SF (control, or treated with 1µM BMS for 7 days). Scale bars indicate 200µm.

(D) SA-βGal was determined 7 or 14 days after drug treatment with 1µM BMS, 10µM αMSH and 10µM [D-Trp^8]-γMSH. Senescence was analyzed by SA-βGal$^+$ staining. Data represent mean values ±SE (n=3-5, one-way ANOVA vs. control).

(E) Melanin synthesis was analyzed on B16-F10 mouse melanocytes treated with 1µM BMS for 4 days using Fontana-Masson staining. Scale bars indicate 1000µm.

(F) B16-F10 melanocytes treated with 1µM BMS for 4 days were centrifuged to visualize pellet colour denoting melanin synthesis. Melanin was further quantified on supernatants by measuring absorbance at 405nm. Data represent mean values ±SE (n=5, Student's t-test vs. control).

(G) SF were stained with Fontana-Masson after treatment with 1µM BMS for 7 days. Scale bar indicate 200µm.

(H) Gene expression of tyrosinase (TYR) was analyzed on SF treated with 1µM BMS or 10µM αMSH. HPRT expression was used as control.

(I) Senescence was assessed by SA-βGal$^+$ staining on BMS (1µM) treated skin fibroblasts. Scale bars indicate 200µm.

(J) Signaling via cAMP (15 min stimulation) and ERK-phosphorylation (5 min stimulation) induced by BMS was studied on HEK293 cells transiently transfected with *MC1R*, *MC3R*, *MC4R* or *MC5R*. Data represent mean values ±SE (n=2).

(K) Cytokines release was evaluated by ELISA on supernatants from senescent SF treated with 1µM BMS for 7 days. Data represent mean values ±SE (n=10, Student's t-test vs. control).

Source data are provided as Supplementary Source Data file.

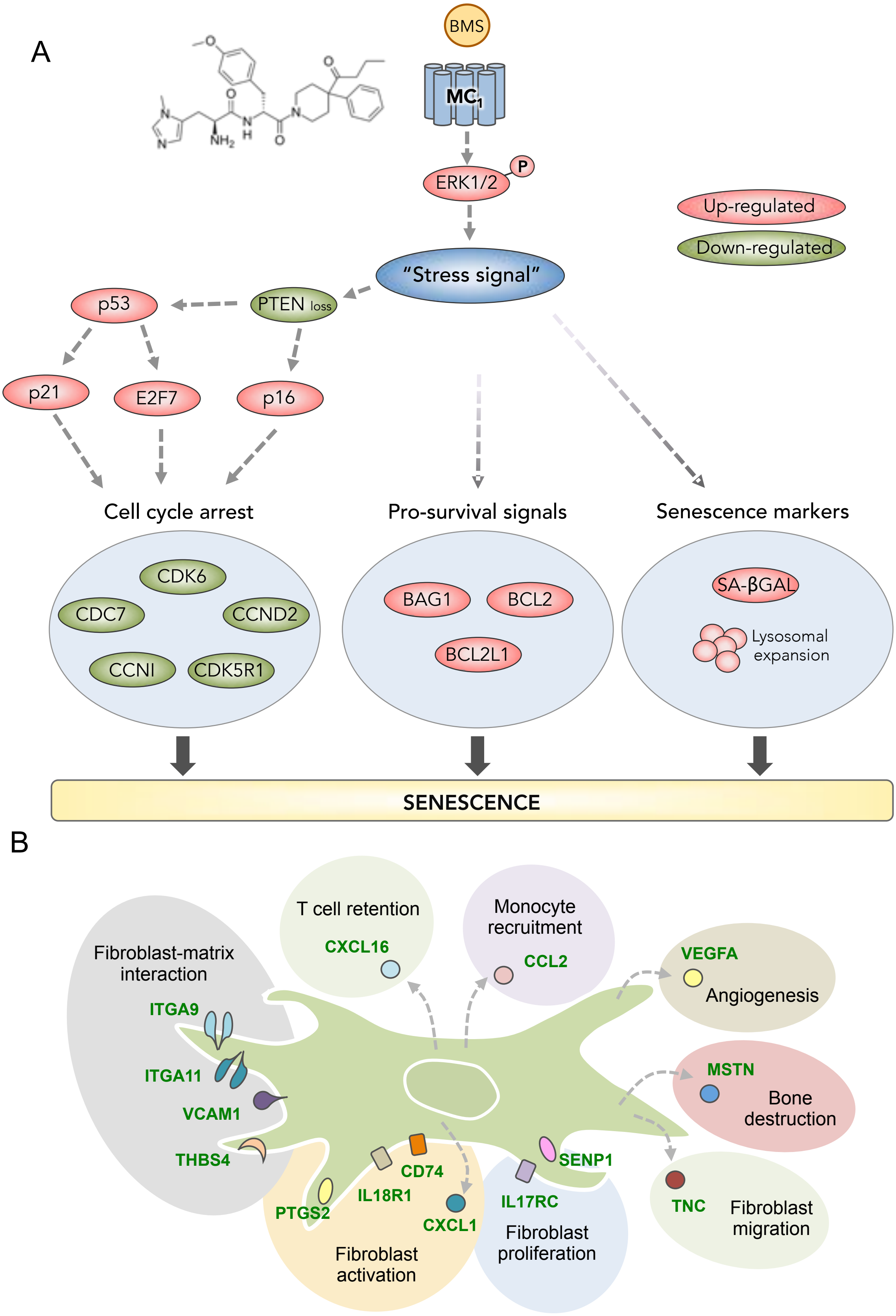

Supplementary Figure 2. Genes identified by RNAseq associated with senescence and SF activation.

(A) Senescence-related genes reported on Figure 3C and other senescence markers identified, such as p53 (by western blot) and SA-βGal (by histochemistry). The chemical structure of BMS-470539 is shown.

(B) Downregulation of genes related to SF activation and aggressiveness reported on Figure 3G.

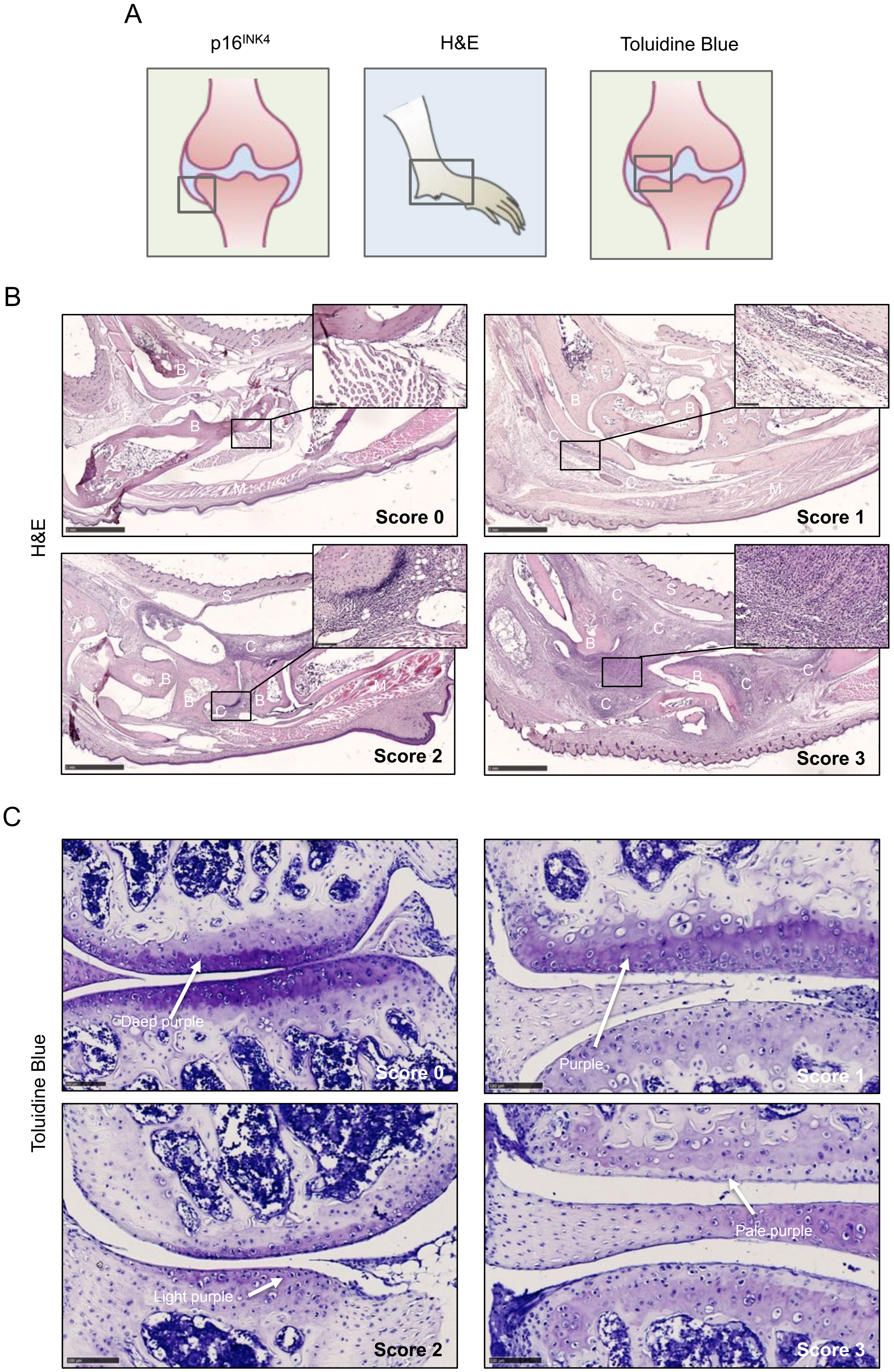
A
p16INK4
H&E
Toluidine Blue
B
H&E
Score 0
Score 1
Score 2
Score 3
C
Toluidine Blue
Deep purple
Purple
Light purple
Pale purple
Score 0
Score 1
Score 2
Score 3

Supplementary Figure 3. Histological analysis on mouse joints.

(A) The location where images for IF and histological analysis on Figure 5 were taken are shown.

(B) Scale used for scoring degree of cell infiltration. Cell infiltration was analysed by H&E staining. Large images were taken at 2.5X magnification and scale bars indicate 1 mm. Small inserts images were taken at 20X magnification and scale bars indicate 100μm. B= bone, C= cell infiltration, F= fat, M= muscle, S= skin.

(C) Scale used for scoring degree of cartilage integrity. Cartilage integrity was analysed by toluidine blue staining. Deep purple colour indicates intact cartilage while gradual loss of colour accounts for loss of sulphated proteoglycans, indicative of cartilage damage. Images were taken at 20X magnification. Scale bars indicate 100μm.

Supplementary Figure 4. Anti-mouse p16^{INK4} antibody validation.

(A) p16^{INK4} expression was assessed by immunofluorescence on knee joints on mice treated with BMS. The Abcam ab54210 (clone 2D9A12) -in red-, was compared to the knock-out validated antibody from the CNIO (clone PABLO33B) -in green-. Scale bars indicate 200µm (20X) and 100µm (40X).

(B) p16^{INK4} expression was assessed by immunofluorescence on 4T1 and NIH 3T3 cells three days after treatment with UV light. Scale bars indicate 200µm.

A

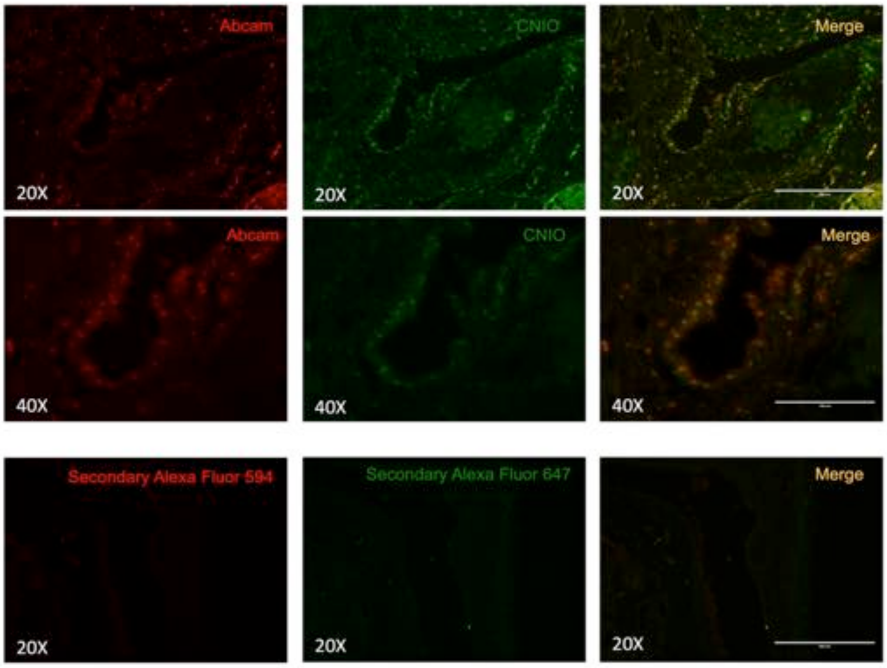

B

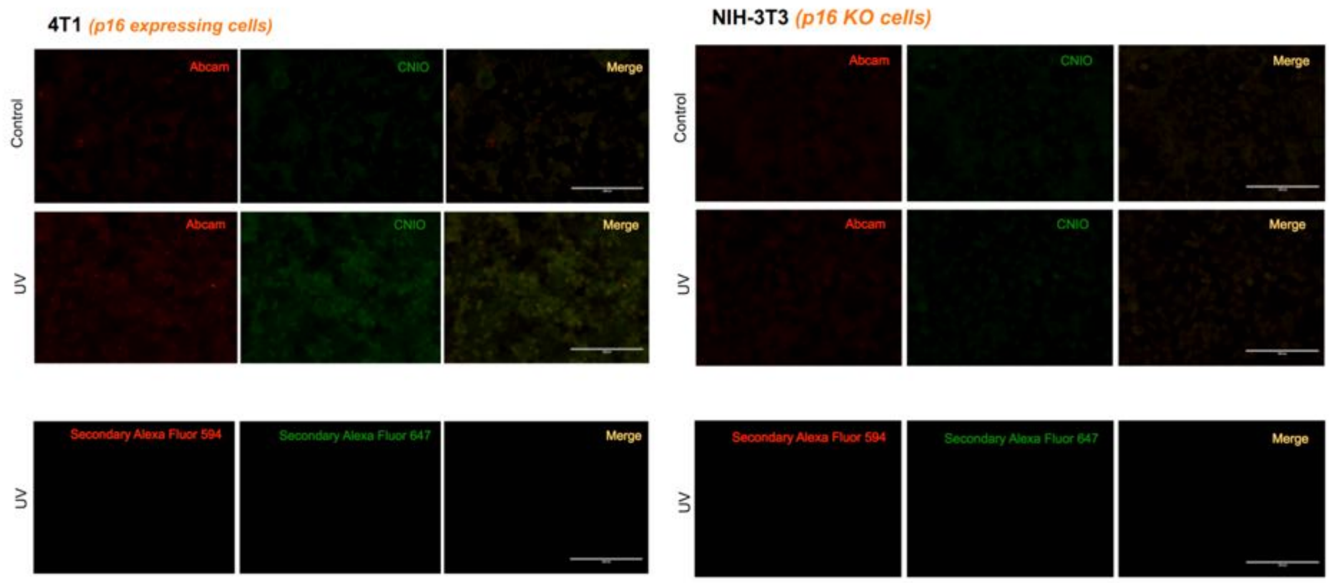

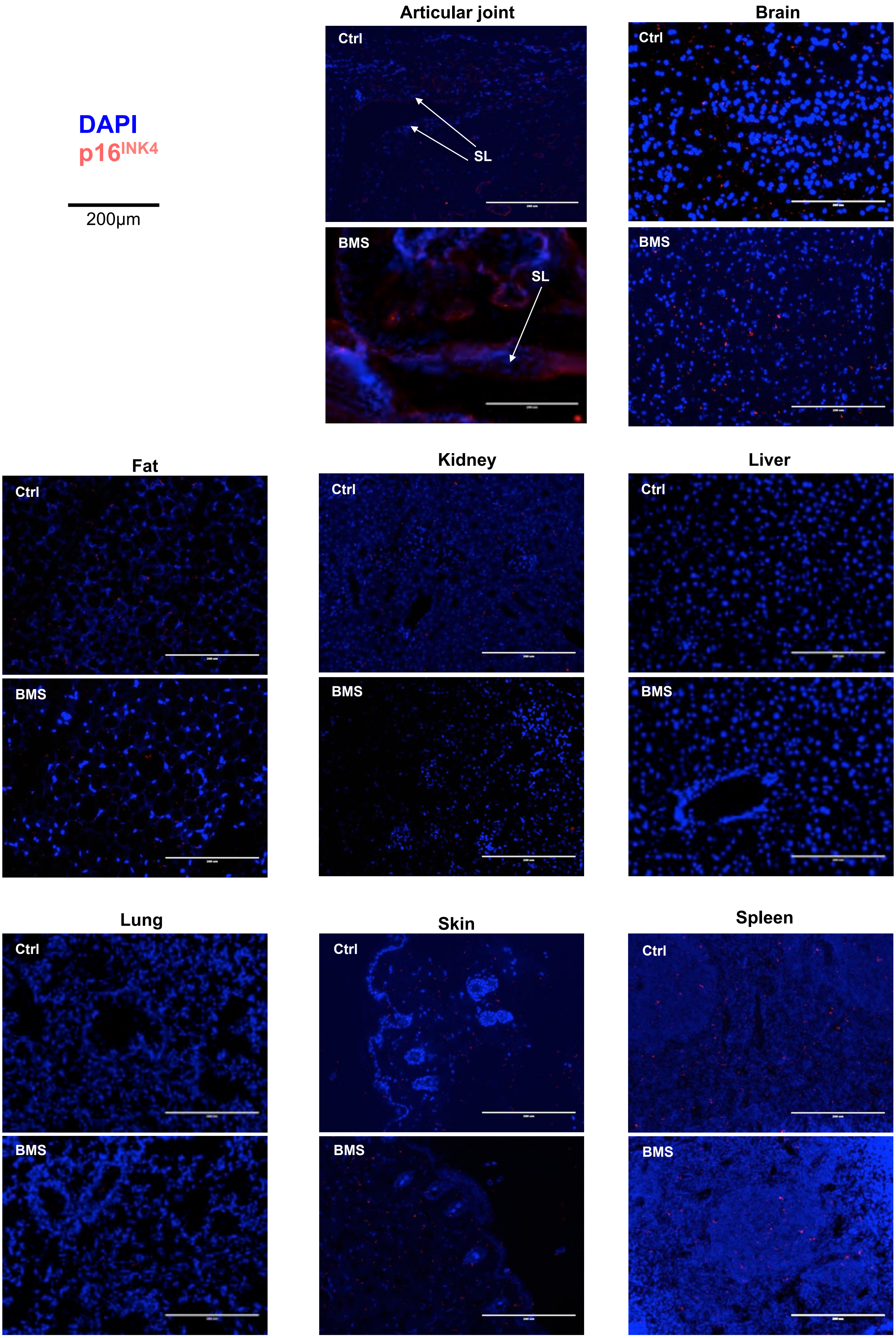

DAPI
p16INK4
200µm
Articular joint
Brain
Ctrl
SL
BMS
SL
Fat
Kidney
Liver
Ctrl
BMS
Lung
Skin
Spleen
Ctrl
BMS

Supplementary Figure 5. Expression of p16^{INK4} on mouse tissues.
To account for off-target effects, p16^{INK4} expression was assessed by immunofluorescence on brain, fat, kidney, liver, lung, skin and spleen on mice treated with 18mg/kg BMS intraperitoneally for 6 days, compared with control (vehicle treated) mice. Tissues were formalin-fixed and embedded in paraffin. Scale bars indicate 200μm.
