# Supplementary material for: Therapeutic senescence via GPCR activation in synovial fibroblasts facilitates resolution of arthritis
Source: Nat Commun. 2020 Feb 6;11:745. doi: 10.1038/s41467-020-14421-x (PMC7005314; doi:10.1038/s41467-020-14421-x)
Supplement: Supplementary file 2 — Reporting Summary [file 41467_2020_14421_MOESM2_ESM.pdf]

nature research

Corresponding author(s): NCOMMS-19-02155

Last updated by author(s): 2019/02/26

Reporting Summary

Nature Research wishes to improve the reproducibility of the work that we publish. This form provides structure for consistency and transparency in reporting. For further information on Nature Research policies, see Authors & Referees and the Editorial Policy Checklist .

Statistics

For all statistical analyses, confirm that the following items are present in the figure legend, table legend, main text, or Methods section.

n/a	Confirmed	
☐	☒	The exact sample size (*n*) for each experimental group/condition, given as a discrete number and unit of measurement
☐	☒	A statement on whether measurements were taken from distinct samples or whether the same sample was measured repeatedly
☐	☒	The statistical test(s) used AND whether they are one- or two-sided<br>*Only common tests should be described solely by name; describe more complex techniques in the Methods section.*
☒	☐	A description of all covariates tested
☒	☐	A description of any assumptions or corrections, such as tests of normality and adjustment for multiple comparisons
☒	☐	A full description of the statistical parameters including central tendency (e.g. means) or other basic estimates (e.g. regression coefficient) AND variation (e.g. standard deviation) or associated estimates of uncertainty (e.g. confidence intervals)
☐	☒	For null hypothesis testing, the test statistic (e.g. *F*, *t*, *r*) with confidence intervals, effect sizes, degrees of freedom and *P* value noted<br>*Give P values as exact values whenever suitable.*
☒	☐	For Bayesian analysis, information on the choice of priors and Markov chain Monte Carlo settings
☒	☐	For hierarchical and complex designs, identification of the appropriate level for tests and full reporting of outcomes
☒	☐	Estimates of effect sizes (e.g. Cohen's *d*, Pearson's *r*), indicating how they were calculated

Our web collection on statistics for biologists contains articles on many of the points above.

Software and code

Policy information about availability of computer code

Data collection	GraphPad Prism v7.0d; SDS 7900HT v2.3; Novostar v1.30 R3; GATC Expression analysis v3.1.4; HTSeqv0.6.1p1;
Data analysis	Microsoft Excel for Mac 2011 V14.7.2 (170228); GraphPad Prism v7.0d; R/Bioconductor v3.5, HISAT2 aligner, HTSeqv0.6.1p1, DAVID v6.8, Panther Classification System v12.0, STRING v10.5., ImageJ v1.43u.

For manuscripts utilizing custom algorithms or software that are central to the research but not yet described in published literature, software must be made available to editors/reviewers. We strongly encourage code deposition in a community repository (e.g. GitHub). See the Nature Research guidelines for submitting code & software for further information.

Data

Policy information about availability of data

All manuscripts must include a data availability statement. This statement should provide the following information, where applicable:

- Accession codes, unique identifiers, or web links for publicly available datasets
- A list of figures that have associated raw data
- A description of any restrictions on data availability

Raw data files for the RNA sequencing analysis have been deposited in the NCBI Gene Expression Omnibus under accession number GEO: GSE98658. Data presented in Figure 3 and Figure 4A-B are related to this raw data.

Field-specific reporting

Please select the one below that is the best fit for your research. If you are not sure, read the appropriate sections before making your selection.

Life sciences study design

All studies must disclose on these points even when the disclosure is negative.

Sample size	The present work started as an exploratory study initially performed in 5 primary synovial fibroblast lines from arthritis patients, and results were confirmed and reproduced in up to 20 patient samples. No statistical method was then used to pre-determine sample size
Data exclusions	No data were excluded from this study
Replication	Replication of the induction of senescence was successful in multiple primary lines (n=20) was proven consistent and reproducible
Randomization	Randomization was not relevant to our study as we were not conducting an interventional study
Blinding	Randomization was not relevant to our study as we were not conducting an interventional study

Reporting for specific materials, systems and methods

We require information from authors about some types of materials, experimental systems and methods used in many studies. Here, indicate whether each material, system or method listed is relevant to your study. If you are not sure if a list item applies to your research, read the appropriate section before selecting a response.

Materials & experimental systems

n/a	Involved in the study	
☐	☒	Antibodies
☐	☒	Eukaryotic cell lines
☒	☐	Palaeontology
☐	☒	Animals and other organisms
☒	☐	Human research participants
☒	☐	Clinical data

Methods

n/a	Involved in the study	
☒	☐	ChIP-seq
☒	☐	Flow cytometry
☒	☐	MRI-based neuroimaging

Antibodies

Antibodies used

1. Anti-CDKN2A/p16INK4a antibody [2D9A12] - Abcam Cat#ab54210
2. Anti-p16INK4 antibody [PABLO33B] - CNIO
3. Anti-p53 antibody [EPR17343] - Abcam Cat#ab179477
4. Cleaved Caspase-3 (Asp175) Polyclonal Antibody - Cell Signaling Cat#9661
5. Anti-NOTCH3 antibody Polyclonal - Abcam Cat#ab23426
6. Goat anti-Mouse IgG (H+L) Alexa Fluor 594 Polyclonal antibody - ThermoFisher Cat#A11032
7. Goat anti-Rabbit IgG (H+L) Alexa Fluor 594 Polyclonal antibody - ThermoFisher Cat#A11072
8. Goat anti-Rabbit IgG (H+L) Cross-Adsorbed Secondary Antibody, Alexa Fluor 647 Polyclonal antibody - ThermoFisher Cat#A11032
9. Goat anti-Rat IgG (H+L) Cross-Adsorbed Secondary Antibody, Alexa Fluor 647 Polyclonal antibody - ThermoFisher Cat#A21247
10. Mouse Monoclonal anti-α-Tubulin [B-5-1-2] - Sigma-Aldrich Cat#T5168
11. Phospho-p44/42 MAPK (Thr202/Tyr204) Rabbit mAb [197G2] - Cell Signaling Cat#4377
12. p44/42 MAPK (Erk1/2) Rabbit mAb [137F5] - Cell Signaling Cat#4695
13. Polyclonal Goat anti-mouse IgG-HRP - Dako Cat#P0447
14. Polyclonal Goat anti-rabbit IgG-HRP - Dako Cat#P0448

Validation

1. https://www.abcam.com/cdkn2ap16ink4a-antibody-2d9a12-ab54210.html
2. https://www.cnio.es/en/research-innovation/services/monoclonal-antibodies/
3. https://www.abcam.com/p53-antibody-epr17343-ab179477.html
4. https://www.cellsignal.co.uk/products/primary-antibodies/cleaved-caspase-3-asp175-antibody/9661
5. https://www.abcam.com/notch3-antibody-ab23426.html
6. https://www.thermofisher.com/antibody/product/Goat-anti-Mouse-IgG-H-L-Highly-Cross-Adsorbed-Secondary-Antibody-Polyclonal/A-11032
7. https://www.thermofisher.com/antibody/product/Goat-anti-Rabbit-IgG-H-L-Cross-Adsorbed-Secondary-Antibody-Polyclonal/A-11072
8. https://www.thermofisher.com/antibody/product/Goat-anti-Mouse-IgG-H-L-Highly-Cross-Adsorbed-Secondary-Antibody-Polyclonal/A-11032

9. https://www.thermofisher.com/antibody/product/Goat-anti-Rat-IgG-H-L-Cross-Adsorbed-Secondary-Antibody-Polyclonal/A-21247
10. https://www.sigmaaldrich.com/catalog/product/sigma/t5168?lang=en&region=GB
11. https://www.cellsignal.co.uk/products/primary-antibodies/phospho-p44-42-mapk-erk1-2-thr202-tyr204-197g2-rabbit-mab/4377
12. https://www.cellsignal.co.uk/products/primary-antibodies/p44-42-mapk-erk1-2-137f5-rabbit-mab/4695
13. https://www.agilent.com/store/en_US/Prod-P044701-2/P044701-2
14. https://www.agilent.com/store/productDetail.jsp?catalogId=P044801-2

Eukaryotic cell lines

Policy information about cell lines

Cell line source(s)	HEK293T -ATCC
Authentication	This cell line was only used for transfection purposes to study the signaling induced by the activation of the receptor transfected. Any aspect of the biology of this cell line was studied and cells were then not authenticated.
Mycoplasma contamination	Cells were negative for mycoplasma contamination
Commonly misidentified lines (See ICLAC register)	None

Animals and other organisms

Policy information about studies involving animals; ARRIVE guidelines recommended for reporting animal research

Laboratory animals	C57BL/6NCrl, male, 6 weeks old; Mc1re/e, male, 6 weeks old; Mc3r-/-, male, 6 weeks old
Wild animals	This study did not involve wild animals
Field-collected samples	This study did not involve samples collected from the field
Ethics oversight	All the animal studies were approved by and performed under the guidelines of the Ethical Committee for the Use of Animals, Barts and The London School of Medicine and Home Office regulations (Guidance on the Operation of ASPA 1986)

Note that full information on the approval of the study protocol must also be provided in the manuscript.
